# Supplementary material for: A fault-intrusion-tolerant system and deadline-aware algorithm for scheduling scientific workflow in the cloud
Source: PeerJ Comput Sci. 2021 Nov 2;7:e747. doi: 10.7717/peerj-cs.747 (PMC8576554; doi:10.7717/peerj-cs.747)
Supplement: Supplemental Information 6 [file peerj-cs-07-747-s006.zip › Myproject1.1/docs/org/cloudbus/cloudsim/power/PowerDatacenter.html]

PowerDatacenter


JavaScript is disabled on your browser.


- Overview
- Package
- Class
- Use
- Tree
- Deprecated
- Index
- Help

- Prev Class
- Next Class

- Frames
- No Frames

- All Classes

- Summary:
- Nested |
- Field |
- Constr |
- Method

- Detail:
- Field |
- Constr |
- Method


org.cloudbus.cloudsim.power

## Class PowerDatacenter

- java.lang.Object
- - org.cloudbus.cloudsim.core.SimEntity
  - - org.cloudbus.cloudsim.Datacenter
    - - org.cloudbus.cloudsim.power.PowerDatacenter

- All Implemented Interfaces:
  :   java.lang.Cloneable

  Direct Known Subclasses:
  :   PowerDatacenterNonPowerAware

  ---

    

  ```
  public class PowerDatacenter
  extends Datacenter
  ```

  PowerDatacenter is a class that enables simulation of power-aware data centers.
  If you are using any algorithms, policies or workload included in the power package please cite
  the following paper:
  Anton Beloglazov, and Rajkumar Buyya, "Optimal Online Deterministic Algorithms and Adaptive
  Heuristics for Energy and Performance Efficient Dynamic Consolidation of Virtual Machines in
  Cloud Data Centers", Concurrency and Computation: Practice and Experience (CCPE), Volume 24,
  Issue 13, Pages: 1397-1420, John Wiley & Sons, Ltd, New York, USA, 2012

  Since:
  :   CloudSim Toolkit 2.0

- - ### Field Summary

    - ### Fields inherited from class org.cloudbus.cloudsim.core.SimEntity

      `FINISHED, HOLDING, RUNNABLE, WAITING`
  - ### Constructor Summary

    Constructors

    Constructor and Description

    `PowerDatacenter(java.lang.String name,
    DatacenterCharacteristics characteristics,
    VmAllocationPolicy vmAllocationPolicy,
    java.util.List<Storage> storageList,
    double schedulingInterval)`

    Instantiates a new datacenter.
  - ### Method Summary

    Methods

    | Modifier and Type | Method and Description |
    | --- | --- |
    | `protected double` | `getCloudletSubmitted()` Checks if is cloudlet submited. |
    | `int` | `getMigrationCount()` Gets the migration count. |
    | `double` | `getPower()` Gets the power. |
    | `protected void` | `incrementMigrationCount()` Increment migration count. |
    | `boolean` | `isDisableMigrations()` Checks if is disable migrations. |
    | `protected boolean` | `isInMigration()` Checks if PowerDatacenter is in migration. |
    | `protected void` | `processCloudletSubmit(SimEvent ev, boolean ack)` Processes a Cloudlet submission. |
    | `protected void` | `processVmMigrate(SimEvent ev, boolean ack)` Process the event for an User/Broker who wants to migrate a VM. |
    | `protected void` | `setCloudletSubmitted(double cloudletSubmitted)` Sets the cloudlet submited. |
    | `void` | `setDisableMigrations(boolean disableMigrations)` Sets the disable migrations. |
    | `protected void` | `setMigrationCount(int migrationCount)` Sets the migration count. |
    | `protected void` | `setPower(double power)` Sets the power. |
    | `protected double` | `updateCloudetProcessingWithoutSchedulingFutureEvents()` Update cloudet processing without scheduling future events. |
    | `protected double` | `updateCloudetProcessingWithoutSchedulingFutureEventsForce()` Update cloudet processing without scheduling future events. |
    | `protected void` | `updateCloudletProcessing()` Updates processing of each cloudlet running in this PowerDatacenter. |

    - ### Methods inherited from class org.cloudbus.cloudsim.Datacenter

      `addFile, checkCloudletCompletion, contains, contains, getCharacteristics, getHostList, getLastProcessTime, getRegionalCisName, getSchedulingInterval, getStorageList, getVmAllocationPolicy, getVmList, predictFileTransferTime, processCloudlet, processCloudletCancel, processCloudletMove, processCloudletPause, processCloudletResume, processCloudletStatus, processDataAdd, processDataDelete, processEvent, processOtherEvent, processPingRequest, processVmCreate, processVmDestroy, registerOtherEntity, setCharacteristics, setLastProcessTime, setRegionalCisName, setSchedulingInterval, setStorageList, setVmAllocationPolicy, setVmList, shutdownEntity, startEntity`
    - ### Methods inherited from class org.cloudbus.cloudsim.core.SimEntity

      `cancelEvent, clone, getEventBuffer, getId, getName, getNextEvent, getNextEvent, getState, numEventsWaiting, numEventsWaiting, pause, run, schedule, schedule, schedule, schedule, scheduleFirst, scheduleFirst, scheduleFirst, scheduleFirst, scheduleFirstNow, scheduleFirstNow, scheduleFirstNow, scheduleFirstNow, scheduleNow, scheduleNow, scheduleNow, scheduleNow, selectEvent, send, send, send, send, sendNow, sendNow, sendNow, sendNow, setEventBuffer, setId, setState, waitForEvent`
    - ### Methods inherited from class java.lang.Object

      `equals, finalize, getClass, hashCode, notify, notifyAll, toString, wait, wait, wait`

- - ### Constructor Detail


    - #### PowerDatacenter

      ```
      public PowerDatacenter(java.lang.String name,
                     DatacenterCharacteristics characteristics,
                     VmAllocationPolicy vmAllocationPolicy,
                     java.util.List<Storage> storageList,
                     double schedulingInterval)
                      throws java.lang.Exception
      ```

      Instantiates a new datacenter.

      Parameters:
      :   `name` - the name
      :   `characteristics` - the res config
      :   `schedulingInterval` - the scheduling interval
      :   `utilizationBound` - the utilization bound
      :   `vmAllocationPolicy` - the vm provisioner
      :   `storageList` - the storage list

      Throws:
      :   `java.lang.Exception` - the exception
  - ### Method Detail


    - #### updateCloudletProcessing

      ```
      protected void updateCloudletProcessing()
      ```

      Updates processing of each cloudlet running in this PowerDatacenter. It is necessary because
      Hosts and VirtualMachines are simple objects, not entities. So, they don't receive events and
      updating cloudlets inside them must be called from the outside.

      **Overrides:**
      :   `updateCloudletProcessing` in class `Datacenter`


    - #### updateCloudetProcessingWithoutSchedulingFutureEvents

      ```
      protected double updateCloudetProcessingWithoutSchedulingFutureEvents()
      ```

      Update cloudet processing without scheduling future events.

      Returns:
      :   the double


    - #### updateCloudetProcessingWithoutSchedulingFutureEventsForce

      ```
      protected double updateCloudetProcessingWithoutSchedulingFutureEventsForce()
      ```

      Update cloudet processing without scheduling future events.

      Returns:
      :   the double


    - #### processVmMigrate

      ```
      protected void processVmMigrate(SimEvent ev,
                          boolean ack)
      ```

      **Description copied from class: `Datacenter`**

      Process the event for an User/Broker who wants to migrate a VM. This PowerDatacenter will
      then send the status back to the User/Broker.

      **Overrides:**
      :   `processVmMigrate` in class `Datacenter`

      Parameters:
      :   `ev` - a Sim\_event object


    - #### processCloudletSubmit

      ```
      protected void processCloudletSubmit(SimEvent ev,
                               boolean ack)
      ```

      **Description copied from class: `Datacenter`**

      Processes a Cloudlet submission.

      **Overrides:**
      :   `processCloudletSubmit` in class `Datacenter`

      Parameters:
      :   `ev` - a SimEvent object
      :   `ack` - an acknowledgement


    - #### getPower

      ```
      public double getPower()
      ```

      Gets the power.

      Returns:
      :   the power


    - #### setPower

      ```
      protected void setPower(double power)
      ```

      Sets the power.

      Parameters:
      :   `power` - the new power


    - #### isInMigration

      ```
      protected boolean isInMigration()
      ```

      Checks if PowerDatacenter is in migration.

      Returns:
      :   true, if PowerDatacenter is in migration


    - #### isDisableMigrations

      ```
      public boolean isDisableMigrations()
      ```

      Checks if is disable migrations.

      Returns:
      :   true, if is disable migrations


    - #### setDisableMigrations

      ```
      public void setDisableMigrations(boolean disableMigrations)
      ```

      Sets the disable migrations.

      Parameters:
      :   `disableMigrations` - the new disable migrations


    - #### getCloudletSubmitted

      ```
      protected double getCloudletSubmitted()
      ```

      Checks if is cloudlet submited.

      Returns:
      :   true, if is cloudlet submited


    - #### setCloudletSubmitted

      ```
      protected void setCloudletSubmitted(double cloudletSubmitted)
      ```

      Sets the cloudlet submited.

      Parameters:
      :   `cloudletSubmitted` - the new cloudlet submited


    - #### getMigrationCount

      ```
      public int getMigrationCount()
      ```

      Gets the migration count.

      Returns:
      :   the migration count


    - #### setMigrationCount

      ```
      protected void setMigrationCount(int migrationCount)
      ```

      Sets the migration count.

      Parameters:
      :   `migrationCount` - the new migration count


    - #### incrementMigrationCount

      ```
      protected void incrementMigrationCount()
      ```

      Increment migration count.


- Overview
- Package
- Class
- Use
- Tree
- Deprecated
- Index
- Help

- Prev Class
- Next Class

- Frames
- No Frames

- All Classes

- Summary:
- Nested |
- Field |
- Constr |
- Method

- Detail:
- Field |
- Constr |
- Method
